# Serum Adropin Levels in Patients with Rheumatoid Arthritis

**DOI:** 10.3390/life12020169

**Published:** 2022-01-24

**Authors:** Petra Simac, Dijana Perkovic, Ivona Bozic, Nada Bilopavlovic, Dinko Martinovic, Josko Bozic

**Affiliations:** 1 Division of Clinical Immunology and Rheumatology, Department of Internal Medicine, University Hospital of Split, 21000 Split, Croatia; petra.simac@mefst.hr (P.S.); ivbozic@kbsplit.hr (I.B.); 2 Department of Medical Laboratory Diagnostics, University Hospital of Split, 21000 Split, Croatia; nbilopav@kbsplit.hr; 3 Department of Pathophysiology, School of Medicine, University of Split, 21000 Split, Croatia; dinko.martinovic@mefst.hr (D.M.); josko.bozic@mefst.hr (J.B.)

**Keywords:** adropin, rheumatoid arthritis, metabolic homeostasis, inflammatory disease

## Abstract

Adropin is a secretory protein that mainly modulates metabolic homeostasis and endothelial function. There is growing evidence supporting association of adropin with various inflammatory diseases, including rheumatoid arthritis (RA). This study aimed to compare serum adropin levels between 70 patients with RA and 70 matched healthy controls. Furthermore, we explored adropin correlations with RA disease activity, glucose metabolism parameters and inflammatory biomarkers. Serum adropin levels were determined by a competitive enzyme-linked immunosorbent assay. Serum adropin levels were significantly lower in RA patients than in the control group (2.85 ± 0.91 vs. 4.02 ± 0.99 ng/mL, *p* < 0.001). In the RA group, serum adropin levels had a significant negative correlation with total cholesterol (r = −0.172, *p* = 0.043), HbA1c (r = −0.406, *p* < 0.001), fasting glucose (r = −0.377, *p* < 0.001) and HOMA-IR (the homeostasis model assessment-estimated insulin resistance; (r = −0.315, *p* = 0.008)). Multiple linear regression analysis showed that serum adropin levels retained a significant association with levels of fasting glucose (β ± SE, −0.450 ± 0.140, *p* = 0.002) and HbA1c (−0.528 ± 0.223, *p* = 0.021) after model adjustments. These findings imply that adropin could have an impact on metabolic homeostasis in RA, although further well-designed studies are warranted in order to establish this.

## 1. Introduction

Rheumatoid arthritis (RA) is the most prevalent inflammatory rheumatic disease that primarily affects synovial tissue leading to joint damage and long-term impairment, and is often burdened with numerous comorbidities. The most important among them are cardiovascular diseases (CVDs) [1]. Major cardiovascular events (CVEs) are the leading causes of death in RA and reduce patient life expectancy [2]. There are several crucial factors contributing to the development of these comorbidities, including the interference of traditional cardiovascular (CV) risk factors (type 2 diabetes (T2D) and high blood pressure (HBP)), in addition to rheumatoid pro-inflammatory ambience [3,4,5,6]. Moreover, it is well established that RA related pro-inflammatory cytokines, tumor necrosis factor alpha (TNF-α), interleukin-1b (IL-1b) and IL-6, have a pivotal role in atherosclerosis and metabolic dysregulation [7]. Furthermore, those cytokines seem to be involved in the pathophysiology of insulin resistance (IR) [8].

Adropin is a secretory peptide consisting of 76 amino acids, and it is encoded by the Energy Homeostasis Associated gene (*ENHO*) [9]. *ENHO* is primarily expressed in the liver and brain, but its presence is also detected in the peripheral blood mononuclear cells, heart, muscles and kidneys [9,10]. Several studies on genetically modified animals have demonstrated a significant correlation of adropin overexpression with improved glucose tolerance, reduced IR (the homeostatic model assessment of insulin resistance–HOMA-IR) and the promotion of carbohydrates in oxidative reactions [10,11]. Apart from these well-investigated roles in glucose homeostasis and lipid metabolism, recent data have highlighted the role of adropin as a potentially prominent regulatory component of the vascular endothelium [9,12]. Some studies have shown that adropin is involved in diverse pathologies, including diabetes mellitus (DM) [13], coronary artery disease [14], arterial hypertension [15], inflammatory bowel disease (IBD) [16] and osteoarthritis (OA) [17]. Interestingly, serum adropin levels in patients with aforementioned diseases were found to be low [13,14,15,16,17]. Moreover, serum adropin levels have been investigated in systemic sclerosis (SSc) and Sjögren’s syndrome (SS), suggesting a potential interrelation between adropin and these autoimmune diseases [18,19].

To the best of our knowledge, only one study compared serum adropin levels with *ENHO* gene expression in a small subset of patients with RA and systemic lupus erythematosus (SLE) with a group of healthy controls. However, they did not find significant differences of serum adropin levels among the study groups [20]. Taking into consideration the role of adropin in glycolipid metabolism [9,12] and its association with inflammatory signaling pathways, including TNF-α and IL-6 [17,21], we hypothesized that adropin may be implicated in the pathophysiology of RA. Hence, the aim of this study was to evaluate the relationship between serum adropin levels and RA disease activity, glucose metabolism parameters and inflammatory biomarkers.

## 2. Materials and Methods

### 2.1. Study Design

This cross-sectional study was performed at the Department of Internal Medicine, Division of Clinical Immunology and Rheumatology, University Hospital of Split from October 2020 to June 2021.

### 2.2. Ethical Considerations

All subjects gave their informed and individually signed consent for inclusion before they participated in the study. The study was approved by the Ethics Committee of University Hospital of Split (Class: 500-03/20-01/109; Registration number: 2181-147-01/06/M.S.-20-02) and was conducted in accordance with all ethical principles of the Seventh Revision of the Helsinki Declaration from 2013.

### 2.3. Subjects

Seventy patients with RA and 70 healthy controls, matched by age and sex, were included in this study. The flow diagram of the study is depicted in the Supplementary Figure S1. Patients with RA were enrolled consecutively from the outpatient clinic of the Department of Clinical Immunology and Rheumatology, University Hospital of Split. The control group consisted of healthy volunteers from the local primary health care center. RA diagnosis was made in accordance with the latest 2010 ACR/EULAR classification criteria [22]. The inclusion criteria were age between 18 and 80 years; RA duration of at least two years; obligatory treatment with biologic or targeted synthetic disease-modifying antirheumatic drugs (DMARDs): TNF-α inhibitors, IL-6 inhibitors, B-cell targeted therapies and Janus kinase (JAK) inhibitors. The exclusion criteria were chronic autoimmune disease other than RA, DM, congestive heart failure, coronary heart disease, chronic kidney disease, respiratory and liver diseases, any kind of malignancy, current pregnancy or breastfeeding, drug abuse, consumption of alcohol more than 40 g/day, current acute or chronic infection and daily oral glucocorticoid therapy ≥10 mg.

### 2.4. Anthropometric Measurements and Clinical Examination

Detailed medical history, a physical examination, anthropometric measurements and laboratory analysis of all the participants were obtained and performed. After collecting clinically relevant data from medical records, we performed systemic and rheumatologic examinations, alongside the assessment of body weight and height, body mass index (BMI), blood pressure and heart ratio. The calibrated medical scale with built-in heights (Seca, Birmingham, UK) was used for measurement of body weight and height and BMI was calculated by dividing the value of body mass (kg) by the squared value of height (m2). A standard mercury sphygmomanometer Riester Big Ben Aneroid (Rudolf Riester GmbH, Jungingen, Germany) was used for measurement of arterial blood pressure. Participants were seated and relaxed for 10 min before the measurements, with the upper arm at heart level, and the mean value of the two measurements taken in account. Finally, data on smoking, coffee and alcohol consumption were checked with all study participants. Furthermore, all subjects showing any symptom of inflammation, malignant, autoimmune or chronic disease, were excluded from the control group.

### 2.5. Disease Severity Assessment and Measurement of Functional Disability

Disease Activity Score-28 (DAS28) and the Stanford Health Assessment Questionnaire (HAQ) were determined only in the patient group for the assessment of RA activity and functional disability, retrospectively. DAS28 score ranges from <2.6 to >5.1. The gradation of RA activity is defined by the thresholds: <2.6 indicates remission, 2.6 to 3.2 indicates low disease activity, 3.2 to 5.1 indicates moderate disease activity, and >5.1 indicates a high disease activity. The assessment of DAS28 was performed by two experienced rheumatology specialists independently using the online Dawn^®^ visual DAS28 calculator. The score is arrived at by using the number of tender and swollen joints, the level of C-reactive protein (CRP (mg/L)) plus the answers to a general health assessment questionnaire (patient assessment of disease activity using a 100 mm visual analogue scale (VAS) with 0 = best, 100 = worst) [23]. Traditionally, HAQ was established for evaluation of difficulties regarding ordinary activities that patients with RA encounter on a daily basis. The HAQ score ranges from 0 to 3, with a score above 1.5 considered a severe disability. The questionnaire is divided into 20 questions in eight categories, including disturbance of movements of both upper and lower extremities [24].

### 2.6. Blood Sampling and Laboratory Analysis

According to the standard laboratory protocols, the same experienced biochemist analyzed all blood samples from participants, which were obtained after 12 h of overnight fasting from the cubital vein via a polyethylene catheter. Serum samples were centrifuged and stored at −80 °C for further analysis, while routine laboratory tests were performed the same day. In addition, the biochemist was blinded to the participants’ group affiliation. Serum adropin levels were determined using commercially available competitive enzyme-linked immunosorbent assay (Phoenix Pharmaceuticals, Burlingame, CA, USA). Analyses were performed according to the manufacturer’s instructions. Declared sensitivity of the assay was 0.3 ng/mL with linearity range of 0.3–8.2 ng/mL. Fasting glucose was measured photometrically using enzymatic method with hexokinase (Roche Diagnostics, Mannheim, Germany) and electrochemiluminescence immunoassay (Roche Diagnostics, Mannheim, Germany) was used to measure insulin levels. HOMA-IR was used as a surrogate measurement of IR. It was calculated according to the following formula = [fasting glucose (mmol/L) × fasting insulin (µU/mL)/[5,22]. The ion-exchange chromatography method on Tosoh G8 analyzer (Tosoh Bioscience, Tokyo, Japan) was used for Hemoglobin A1c (HbA1c) measurement. Anti-cyclic citrullinated peptide (anti-CCP) antibody levels were determined by chemiluminescence microparticle immunoassay on an Architect analyzer (Abbott, Abbott Park, IL, USA). hsCRP (high-sensitivity CRP) and rheumatoid factor (RF) were determined by the immunoturbidimetric methods (Roche Diagnostics, Mannheim, Germany). RF and anti-CCP were measured only in RA patients. Other biochemical analyses were performed using standard laboratory procedures.

### 2.7. Statistical Analysis

Analyses of the data were performed using MedCalc (MedCalc Software, Ostend, Belgium, version 19.1.2). Quantitative data are presented as the mean ± standard deviation or median (interquartile range), whereas qualitative data were expressed as whole numbers (N) with percentages (%). Normality of distribution was assessed using the Kolmogorov–Smirnov test. Independent samples *t*-test or Mann–Whitney U test were used for comparison of groups in terms of quantitative variables, while the chi-squared (χ2) test was used to determine differences between groups in terms of qualitative variables. Pearson’s correlation or Spearman’s rank correlation were used for the estimation of correlations between variables. Finally, a multiple linear regression analysis was used to determine significant and independent associations of the adropin serum levels, which was defined as a dependent variable. The level of statistical significance was set at a *p*-value < 0.05.

### 2.8. Sample Size Analysis

Sample size analysis was conducted using the data from a pilot study on 10 subjects from the RA population and 10 matched control subjects. The value of serum adropin, which was the main result of the study, was used for the calculation. The mean serum adropin levels were 3.01 ± 1.02 ng/mL in the RA group and 3.77 ± 1.12 ng/mL in the control group. With a type I error of 0.05, and a power of 90%, the required sample size was 43 participants per group.

## 3. Results

### Baseline Characteristics and Laboratory Parameters

There were no statistically significant differences in baseline characteristics between the RA group and the control group (Table 1). In the RA group, 46 (65.7%) subjects had a positive RF and 51 (72.8%) subjects had a positive anti-CCP. Moreover, the mean duration of RA was 15.0 (10.0–20.0) years (Table 1).

Laboratory analyses showed that the RA group, in comparison to the control group, had significantly higher levels of hsCRP (1.7 (0.7–3.1) vs. 1.2 (0.6–1.9) mg/L, *p* = 0.009), serum creatinine (70.0 ± 17.3 vs. 64.1 ± 10.7 μmol/L, *p* = 0.017), total cholesterol (5.3 ± 1.2 vs. 4.7 ± 1.0 mmol/L, *p* = 0.002), LDL cholesterol (3.3 (2.5–3.8) vs. 2.7 (2.0–3.6) mmol/L, *p* = 0.025), fasting glucose (5.0 ± 0.7 vs. 4.7 ± 0.5 mmol/L, *p* = 0.007) and HOMA-IR (2.7 ± 1.6 vs. 2.1 ± 1.1, *p* = 0.027). There were no other significant differences between the RA and the control group in laboratory parameters (Table 2).

**Figure 1:**
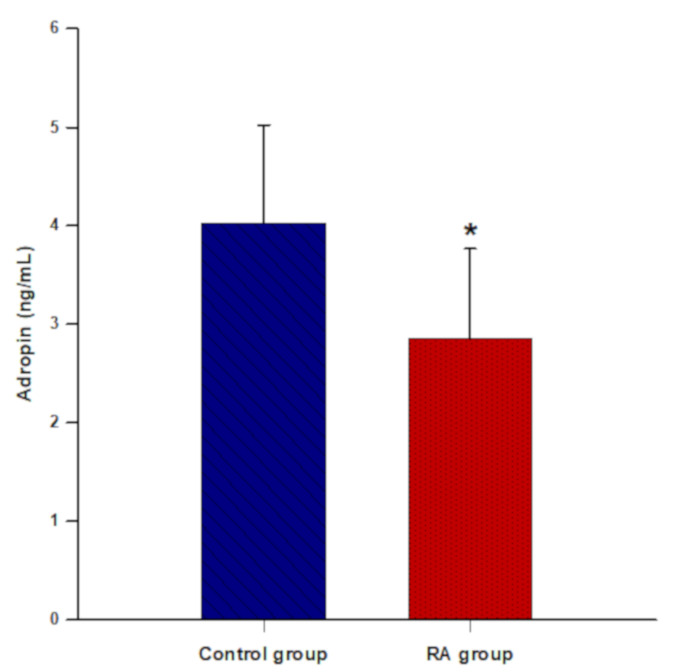
Comparison of serum adropin levels between the control group (*n* = 70) and the RA group (*n* = 70). The data are presented as the mean ± standard deviation. * *t*-test for independent samples (*p* < 0.001).

Serum adropin levels were significantly lower in patients with RA than in the control group (2.85 ± 0.91 vs. 4.02 ± 0.99 ng/mL, *p* < 0.001) (Figure 1).

**Figure 2:**
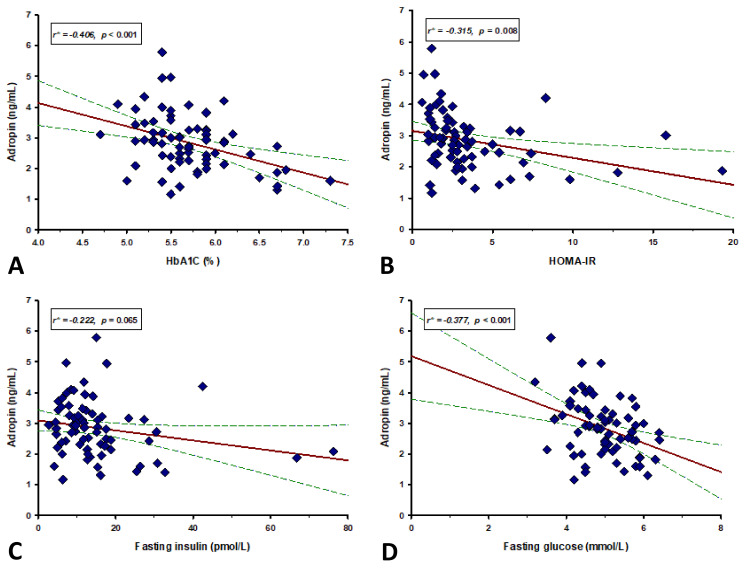
Correlation analysis of adropin levels with HbA1c (**A**), HOMA-IR (**B**), fasting insulin (**C**) and fasting glucose (**D**) in RA group. Red lines represent the regression line, while green lines represent 95% confidence interval. * Pearson’s correlation coefficient.

Furthermore, serum adropin levels had a significant negative correlation with total cholesterol (r = −0.172, *p* = 0.043), HbA1c (r = −0.406, *p* <0.001), fasting glucose (r = −0.377, *p* <0.001) and HOMA-IR (r = −0.315, *p* = 0.008) (Table 3, Figure 2). There were no other significant correlations between serum adropin levels and anthropometric, laboratory and clinical parameters (Table 3).

**Figure 3:**
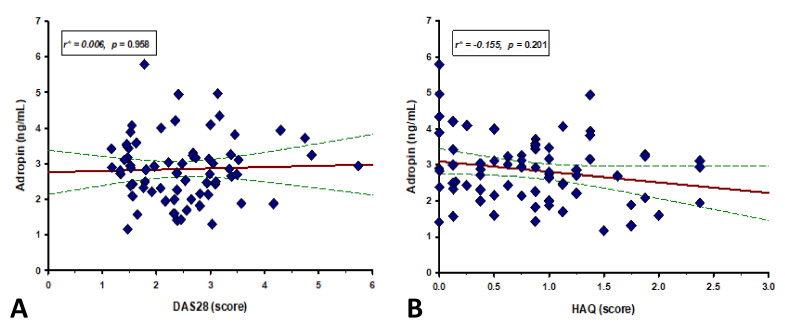
Correlation between serum adropin levels and (**A**) HAQ and (**B**) DAS28 score (*n* = 70). Red lines represent the regression line, while green lines represent 95% confidence interval. * Spearman’s correlation coefficient.

We did not find a significant correlation of serum adropin levels with DAS28 (r = 0.006, *p* = 0.958) and HAQ score (r = −0.155, *p* = 0.201) (Figure 3).

Multiple linear regression analysis showed that serum adropin levels retained significant association with fasting glucose levels (β ± SE, −0.450 ± 0.140, *p* = 0.002) and HbA1c (−0.528 ± 0.223, *p* = 0.021) after model adjustment for age, BMI, DAS28 and HAQ scores and disease duration, with serum adropin levels as a dependent variable (Table 4).

## 4. Discussion

Our study has shown that patients with RA had significantly lower levels of serum adropin than healthy control subjects. In addition, a significant negative correlation was found between serum adropin levels and serum levels of fasting glucose, HbA1c and HOMA-IR values in RA patients, which could emphasize the possible interaction of adropin and energy homeostasis in RA.

As mentioned, only one study has measured serum adropin levels in a small sample of patients with RA and SLE in comparison to healthy controls. Contrary to our results, they did not find any significant difference among the groups, although *ENHO* expression was higher in the RA group. Our study differs from the above-noted in smaller sample size, and exclusion criteria. In addition, they compared serum adropin levels in patients with RA and SLE with the same control group, despite substantial heterogeneity between the groups. Furthermore, their patients were significantly younger and RA duration was shorter, both factors that could interfere with serum adropin levels. [20].

Low levels of circulating adropin have been reported in some disorders with low-grade chronic inflammation, such as T2D [25], atherosclerosis [26], IBD [16], and in patients on hemodialysis [27]. Considering that RA is a systemic inflammatory disease, it was reasonable to expect similar lower serum levels of adropin in patients with RA. The results of our study are in concordance with this. Several various in vitro studies have shown adropin’s multiple anti-inflammatory effects, emphasizing an association of adropin deficiency with the imbalance of the immune cells and inflammatory cytokines [28,29,30,31]. However, even though these findings suggest that adropin has a pivotal immunomodulatory role, the specific underlying mechanisms have not been fully elucidated [29]. The increased production of nitric oxide (NO) parameters in RA, catalyzed by inducible nitric oxide synthase (iNOS), is a direct consequence of the activation of IL-1, TNF-α and interferon-γ (IFN-γ) in macrophages and vascular endothelial cells [21,29]. One of the possible mechanisms is the upregulation of endothelial nitric oxide synthase (eNOS) kinase activity, a process which could be involved in the cascade of RA pathophysiological pathways. This hypothesis is supported by data demonstrating that adropin modifies eNOS activity by activation of vascular endothelial growth factor receptor 2 (VEGFR2). Consequently, the activation of PI3K-Akt and ERK1/2 signaling pathways occurs, with both of them involved in the pathogenesis of RA [32]. Moreover, the leukocyte extravasation and motion process regulated by TNF-α could be disrupted by adropin, thereby showing its anti-inflammatory effects [33]. Regarding these in vitro data, the potential link between adropin and various rheumatic diseases has been investigated [17,18,19,20]. Increased serum adropin levels have been observed in patients with pSS, SSc and Behcet’s disease (BD) [18,19], whereas serum adropin was reduced in patients with OA [17]. The latter study has demonstrated a negative correlation of serum adropin levels with TNF-α and inflammatory cells in patients with knee OA. These results implicate a connection between adropin and markers of inflammation [17]. Despite the conflicting results of these studies, the interference of adropin in inflammatory signaling pathways in systemic autoimmune diseases remains plausible. Consistent with the fact that the primary pro-inflammatory cytokine in RA is TNF-α, the lower adropin values found in our patients could be explained in the context of the results in OA. Yet, it is important to address that our patients have been on therapy with biologics and targeted small molecules that at least partially neutralize TNF-α, thus possibly affecting serum adropin levels. For future perspectives, a multicenter longitudinal study with naïve RA patients should evaluate all of these findings.

The precise role of adropin and *ENHO* expression in inflammatory rheumatic diseases still needs to be clarified. Gregersen et al. reported a genetic variant in *ENHO* expression in RA, but it has not been established whether it affects serum adropin levels [34]. Moreover, Yolbas et al. demonstrated a higher *ENHO* expression in RA patients in the absence of upstreamed serum levels of adropin, suggesting that adropin could be associated with pannus formation in RA. This hypothesis was additionally explained by the lack of pannus formation in patients with SLE and healthy controls [20]. One of the most important regulators of the genomic transcription of chemokines, cytokines, and adhesion molecules is the kappa B nuclear transcription factor (NF-kB) [35]. Interestingly, an increased level of NF-kB was found in pannus of both patients and animal models with RA, emphasizing its role in active inflammation in RA [36,37,38]. These findings could indicate that NF-kB, through molecular imbalance, leads to alteration of *ENHO* expression and, consequently serum adropin levels. This could be one of the possible pathophysiological mechanisms by which pro-inflammatory cytokines (TNF-α, IL-1 and IL-6) participate in *ENHO* expression and serum adropin levels in RA. A recently published study has shown that *ENHO* expression is mediated via members of the nuclear receptor (NR) 1F subfamily, RORα and RORγ genes [39]. An animal study conducted on RORα-deficient Stagger mice that were fed a high-fat diet showed increased mRNA levels of TNF-α, IL-1β, and IL-6 [40,41]. Furthermore, RORα negatively regulates the cytokine-induced inflammatory response by impeding the NF-κB signaling pathway [42]. The link between RORα and NF-κB could be a potential molecular basis that connects pro-inflammatory cytokines, *ENHO* expression and adropin levels in RA, but further studies are needed to explore these theses. On the other hand, adropin deficiency results in elevated expressions of inflammation-related genes (IL-1b, IL-6 and TNF-α) in adropin knockout mice (AdrKO) mice [43]. Similar to this in vitro finding, it was proposed that the levels of adropin are in reverse association with the above-mentioned inflammation-related genes in various inflammation states [29]. These results indicate that levels of pro-inflammatory cytokines have influence on the serum adropin levels, and even *ENHO* expression, but more in vivo experiments are required to further confirm these data.

It is well-established that patients with RA are at an increased risk of developing DM due to several factors, most prominently chronic inflammation, glucose intolerance and IR [44,45,46]. IR in RA is largely due to systemic inflammation induced by pro-inflammatory cytokines, particularly TNF-α [44,46]. TNF-α triggers key steps in insulin signaling pathways contributing to the increased IR in patients with RA [46,47]. The significant finding of our study is a negative correlation of serum adropin levels with fasting glucose and HOMA-IR, in concordance with adropin’s role in glucose metabolism [10,11]. It has been shown that lower blood adropin levels were associated with obesity and IR in mice [10]. Moreover, the treatment of obese mice with exogenous adropin resulted in improvement of insulin sensitivity and glucolipid metabolism, with lower expression of lipogenic genes in the liver [10]. Adropin regulates the expression of some of the key adipokines, such as adiponectin, resistin and visfatin [48]. Although, the importance of adipokines in the complex pathophysiology of RA has been well studied, their specific role in the progression of disease remains to be evaluated [49]. Resistin and visfatin have been shown to be connected with both chronic inflammation and bone destruction in RA [50]. Since adropin acts as a regulator of resistin and visfatin expression, it could be considered as a potential anti-inflammatory factor in RA. Similar to our results, a negative correlation between serum adropin levels and levels of fasting glucose and HbA1c and values of HOMA-IR was reported in the study conducted in patients with obstructive sleep apnea. That negative correlation was explained by the possibility of adropin interactions with eNOS and pro-inflammatory cytokines (TNF-α and IL-6) [33]. Therefore, this could be a mechanism by which adropin interferes with RA inflammation.

We did not find a significant correlation between serum adropin levels and DAS28 and HAQ scores, RA duration and hsCRP levels. Based on our findings of negative correlation between serum adropin levels and fasting glucose, HbA1c and HOMA-IR values in RA patients, we can speculate that adropin is somehow involved in metabolic disorders in RA.

Our study has several limitations. It was conducted in a single-center and it had a relatively small sample size. Moreover, it was not possible to completely eliminate all the confounding effects that could interfere with the results of the study. One of them is the possible effects of biological and targeted small molecule therapy on serum adropin levels in our RA study group, due to partial neutralization of TNF-α. Additionally, the cross-sectional design of the study does not allow for definition of any causal relations.

## 5. Conclusions

In conclusion, this is the first study that reported decreased serum adropin levels in patients with RA. Furthermore, adropin was negatively correlated with fasting glucose, HbA1c and HOMA-IR values. These results imply that adropin could have an impact on metabolic homeostasis in RA, although these results should be interpreted cautiously and further larger scale studies are necessary to address them.

## Figures and Tables

**Table 1:** Baseline characteristics of the RA group and the control group.

Parameter	RA Group (*n* = 70)	Control Group (*n* = 70)	*p* *
Female sex (N,%)	64 (91.4)	61 (87.1)	0.737
Age (years)	55.9 ± 12.5	52.5 ± 14.1	0.134
Body weight (kg)	73.2 ± 13.6	69.6 ± 14.1	0.124
Body height (cm)	169.1 ± 7.2	168.1 ± 10.1	0.487
Body mass index (kg/m^2^)	25.6 ± 4.2	25.0 ± 4.3	0.394
SBP (mmHg)	131.2 ± 17.5	126.3 ± 15.2	0.078
DBP (mmHg)	80.9 ± 10.9	78.7 ± 11.4	0.244
Smoking (N,%)	22 (31.4)	16 (22.9)	0.349
Disease duration (years) ^†^	15.0 (10.0–20.0)	-	-
Rheumatoid factor (N,%)	46 (65.7)	-	-
Anti-CCP (N,%)	51 (72.8)	-	-
DAS28 (score) ^‡^	2.49 ± 0.94	-	-
HAQ (score)	0.83 ± 0.60	-	-
csDMARD	50 (71.4)	-	-
tsDMARD	10 (14.2)	-	-
bDMARD	60 (85.8)	-	-

Abbreviations: SBP, systolic blood pressure; DBP, diastolic blood pressure; anti-CCP, anti-cyclic citrullinated peptide antibodies; DAS28, Disease Activity Score 28; HAQ, Health Assessment Questionnaire; scDMARD, conventional synthetic disease-modifying antirheumatic drug; tsDMARD, targeted synthetic disease-modifying antirheumatic drug; bDMARD, biologic disease-modifying antirheumatic drug. Data are presented as the whole number (percentage), the mean ± standard deviation or median (IQR). * chi-square test or *t*-test for independent samples. ^†^ time period since the initial diagnosis. ^‡^ calculated using the hsCRP values.

**Table 2:** Laboratory parameters of the RA group and the control group.

Parameter.	RA Group (*n* = 70)	Control Group (*n* = 70)	*p* *
Erythrocytes (×10^12^/L)	4.4 ± 0.4	4.5 ± 0.5	0.330
Hemoglobin (g/L)	133.8 ± 12.9	136.1 ± 10.8	0.269
TSH (mIU/mL)	2.2 (1.3–3.4)	1.8 (1.2–3.2)	0.365
Urea (mmol/L)	5.2 ± 1.7	5.0 ± 1.6	0.355
Creatinine (μmol/L)	70.0 ± 17.3	64.1 ± 10.7	0.017
hsCRP (mg/L)	1.7 (0.7–3.1)	1.2 (0.6–1.9)	0.009
Triglycerides (mmol/L)	1.4 ± 0.7	1.3 ± 0.6	0.384
Total cholesterol (mmol/L)	5.3 ± 1.2	4.7 ± 1.0	0.002
HDL cholesterol (mmol/L)	1.8 ± 0.4	1.8 ± 0.5	0.515
LDL cholesterol (mmol/L)	3.3 (2.5–3.8)	2.7 (2.0–3.6)	0.025
Fasting glucose (mmol/L)	5.0 ± 0.7	4.7 ± 0.5	0.007
Fasting insulin (pmol/L)	80.3 ± 37.0	71.1 ± 36.2	0.164
HbA1c (%)	5.7 ± 0.4	5.5 ± 0.4	0.052
HOMA-IR	2.7 ± 1.6	2.1 ± 1.1	0.027

Abbreviations: TSH, thyroid stimulating hormone; hsCRP, high sensitivity C-reactive protein; HbA1c, hemoglobin A1c; HOMA-IR, homeostatic model assessment for insulin resistance. Data are presented as the mean ± standard deviation and median (IQR). * *t*-test for independent samples or Mann–Whitney U test.

**Table 3:** Correlation analysis between serum adropin levels and different biochemical, anthropometric and clinical parameters in the RA group (*n* = 70).

Parameter	r *	*p*
hsCRP (mg/L)	0.103 ^†^	0.641
Triglycerides (mmol/L)	−0.199	0.098
Total cholesterol (mmol/L)	−0.172	0.043
HDL (mmol/L)	0.045	0.597
LDL (mmol/L)	−0.057 ^†^	0.641
Urea (mmol/L)	−0.112	0.187
Creatinine (μmol/L)	−0.102	0.230
TSH (mIU/mL)	−0.187 ^†^	0.120
Age (years)	−0.036	0.671
Body mass index (kg/m^2^)	0.026	0.760
SBP (mmHg)	−0.109	0.199
DBP (mmHg)	−0.038	0.659
HAQ (score)	−0.155	0.201
DAS28 (score)	0.006	0.958

Abbreviations: hsCRP, high sensitivity C-reactive protein; TSH, thyroid stimulating hormone; 25OHD, 25 hydroxyvitamin D; SBP, systolic blood pressure; DBP, diastolic blood pressure; HAQ, Health Assessment Questionnaire; DAS28, Disease Activity Score−28. * Pearson’s correlation coefficient. ^†^ Spearman’s rank correlation coefficient.

**Table 4:** Multiple linear regression model of independent predictors for serum adropin levels.

Variable	β ^†^	SE ^‡^	*t*-Value	*p*
Age (years)	−0.007	0.009	−0.816	0.418
Body mass index (kg/m^2^)	0.046	0.024	1.892	0.063
DAS28 (score)	−0.089	0.137	−0.655	0.515
HAQ (score)	0.362	0.208	1.740	0.087
Disease duration (years)	0.014	0.011	1.264	0.211
Fasting glucose (mmol/L)	−0.450	0.140	−3.219	0.002
HbA1c (%)	−0.528	0.223	−2.361	0.021

Abbreviations: DAS28, Disease Activity Score-28; HAQ, Health Assessment Questionnaire; HbA1c, hemoglobin A1c. ^†^ unstandardized coefficient β ^‡^ standard error.

## Data Availability

Data are available on request to corresponding author.

## Supplementary Materials

The following supporting information can be downloaded at: https://www.mdpi.com/article/10.3390/life12020169/s1, Figure S1. The flow diagram of the study.
