# Supplementary material for: Serum Adropin Levels in Patients with Rheumatoid Arthritis
Source: Life (Basel). 2022 Jan 24;12(2):169. doi: 10.3390/life12020169 (PMC8875108; doi:10.3390/life12020169)
Supplement: Supplementary file 1 [file life-12-00169-s001.zip › life-1547153-supplementary.pdf]

Supplementary Materials: Figure S1. The flow diagram of the study.

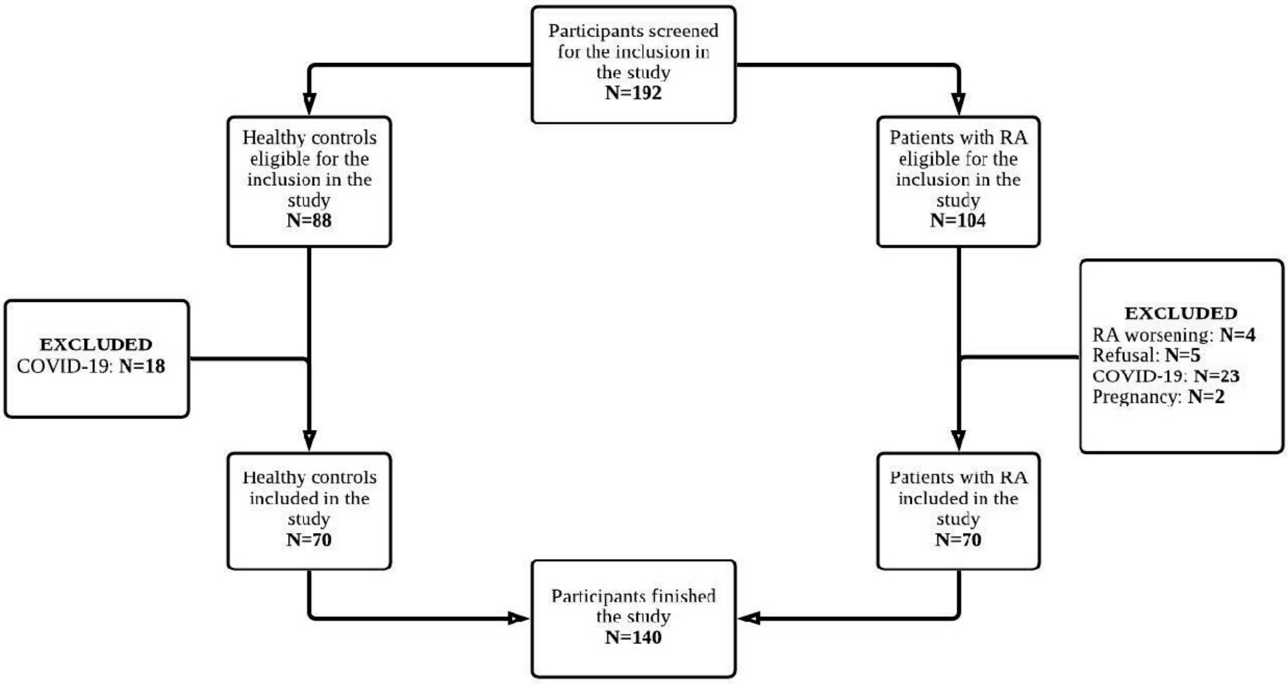

Abbreviations: HCs, healthy controls; RA, rheumatoid arthritis; COVID-19, Coronavirus disease 2019.
